# Molecular characterization and genotype distribution of thioester-containing protein 1 gene in *Anopheles gambiae* mosquitoes in western Kenya

**DOI:** 10.1186/s12936-022-04256-w

**Published:** 2022-08-10

**Authors:** Shirley A. Onyango, Kevin O. Ochwedo, Maxwell G. Machani, Julius O. Olumeh, Isaiah Debrah, Collince J. Omondi, Sidney O. Ogolla, Ming-Chieh Lee, Guofa Zhou, Elizabeth Kokwaro, James W. Kazura, Yaw A. Afrane, Andrew K. Githeko, Daibin Zhong, Guiyun Yan

**Affiliations:** 1 Department of Zoological Sciences, School of Science and Technology, Kenyatta University, Nairobi, Kenya; 2 Sub-Saharan Africa International Centre of Excellence for Malaria Research, Homa bay, Kenya; 3 Centre for Global Health Research, Kenya Medical Research Institute, Kisumu, Kenya; 4 Program in Public Health, College of Health Sciences, University of California at Irvine, Irvine, CA 92697 USA; 5 Department of Biology, Faculty of Science and Technology, University of Nairobi, Nairobi, Kenya; 6 Department of Medical Microbiology, Medical School, University of Ghana, University of Ghana, Accra, Ghana; 7 Department of Biochemistry, Cell and Molecular Biology, West Africa Centre for Cell Biology of Infectious Pathogen, University of Ghana, Accra, Ghana; 8 Center for Global Health and Diseases, Case Western Reserve University, LC 4983, Cleveland, OH 44106 USA

**Keywords:** *Anopheles gambiae*, Thioester-containing protein 1, Genetic diversity, Population structure, Signature of selection, Malaria transmission

## Abstract

**Background:**

Evolutionary pressures lead to the selection of efficient malaria vectors either resistant or susceptible to *Plasmodium* parasites. These forces may favour the introduction of species genotypes that adapt to new breeding habitats, potentially having an impact on malaria transmission. Thioester-containing protein 1 (TEP1) of *Anopheles gambiae* complex plays an important role in innate immune defenses against parasites. This study aims to characterize the distribution pattern of TEP1 polymorphisms among populations of *An. gambiae *sensu lato (*s.l*.) in western Kenya.

**Methods:**

*Anopheles gambiae* adult and larvae were collected using pyrethrum spray catches (PSC) and plastic dippers respectively from Homa Bay, Kakamega, Bungoma, and Kisumu counties between 2017 and 2020. Collected adults and larvae reared to the adult stage were morphologically identified and then identified to sibling species by PCR. TEP1 alleles were determined in 627 anopheles mosquitoes using restriction fragment length polymorphisms-polymerase chain reaction (RFLP-PCR) and to validate the TEP1 genotyping results, a representative sample of the alleles was sequenced.

**Results:**

Two TEP1 alleles (TEP1*S1 and TEP1*R2) and three corresponding genotypes (*S1/S1, *R2/S1, and *R2/R2) were identified. TEP1*S1 and TEP1*R2 with their corresponding genotypes, homozygous *S1/S1 and heterozygous *R2/S1 were widely distributed across all sites with allele frequencies of approximately 80% and 20%, respectively both in *Anopheles gambiae* and *Anopheles arabiensis.* There was no significant difference detected among the populations and between the two mosquito species in TEP1 allele frequency and genotype frequency. The overall low levels in population structure (*F*_ST_ = 0.019) across all sites corresponded to an effective migration index (Nm = 12.571) and low Nei’s genetic distance values (< 0.500) among the subpopulation. The comparative fixation index values revealed minimal genetic differentiation between species and high levels of gene flow among populations.

**Conclusion:**

Genotyping TEP1 has identified two common TEP1 alleles (TEP1*S1 and TEP1*R2) and three corresponding genotypes (*S1/S1, *R2/S1, and *R2/R2) in *An. gambiae s.l.* The TEP1 allele genetic diversity and population structure are low in western Kenya.

**Supplementary Information:**

The online version contains supplementary material available at 10.1186/s12936-022-04256-w.

## Background

*Anopheles gambiae* mosquitoes are competent vectors for malaria in sub-Saharan Africa [1, 2] Ongoing vector control interventions [3, 4] climate change [5–9] and environmental modifications may select vector genotypes or species that adapt to new breeding habitats. These factors may cause vectorial rearrangement exerting selection pressure that could change TEP1 allele frequencies and subsequently, efficient vectors could thrive and continue transmitting malaria. Despite the increased vector densities, malaria transmission is dependent on infectious parasites and competent vectors to influence susceptibility to infections in local vector populations. A vector’s susceptibility and/or resistance to *Plasmodium* parasites is a determining factor for vector competence and is in part influenced by the thioester containing protein 1 (TEP1).

In *Anopheles gambiae,* TEP1 exhibits allelic variations that alter vector competence and subsequently influence malaria infectivity [10, 11]. These variations may be as a result of selective pressures such as climate change and vector control interventions acting on the TEP1 gene that eventually influence the vector's ability to transmit the *Plasmodium* parasite [11]. The TEP1 gene was reported to target the *Plasmodium* parasite in the early stages of infection in the mosquito host mostly the ookinetes [12, 13] either by melanization or lysis [14, 15] effectively reducing oocysts and sporozoite numbers in the vector. However, there is a lack of knowledge on how these allelic polymorphisms in vector competence affect malaria transmission [16, 17]. Furthermore, the distribution of the TEP1 allele in western Kenya regions with varying malaria transmission intensities is unknown. Therefore, understanding molecular mechanisms underlying mosquito genotypes and *Plasmodium* adaptations to different *Anopheles* species is important and could be used to monitor infection trends in vectors that directly have an impact on malaria transmission.

The complement-like thioester-containing protein 1 (TEP1) plays a key role in immunity against pathogens [17–20]. TEP1 is a highly polymorphic protein [21–23] located in the thioester domain (TED) on chromosome 3L coding for 1338 amino acids long protein contributing to phenotypic divergence and demonstrates genetic variations associated with distinct genotypes in its refractoriness to *Plasmodium* parasites. Six allelic classes; TEP1*S1, TEP1*S2, TEP1*S3, TEP1*R1, TEP1*R2, and TEP1*R3 have recently been characterized in the *An. gambiae* complex in Africa [13, 14, 24]. TEP1*S1 and TEP1*R2 are the most common TEP1 alleles identified across Africa. The TEP1*S1 however lacks a defined geographical structure. The TEP1*S2 allele identified in the 4Arr strain is specific to *Anopheles coluzzii* [24] and gets rid of the damaged sperm cells in the male mosquitoes [25] bringing forth varying *Anopheles* population abundance. TEP1*S3 allele closely related to TEP1*S1 is fixed in the G3 strain associated with susceptibility of infection to *Plasmodium berghei* [13]. TEP1*R1 identified in the L3-5 strain depicts the highest level of resistance to *Plasmodium* associated with melanization [13, 25, 26] and documented in *An. coluzzii* in West Africa [24]. A newly identified allele TEP1*R3 is specific to the saline water mosquito, *Anopheles merus* found at the Kenyan coast. Selective pressures influence these variations in the genetic structure of the natural *An. gambiae* populations in different ecological settings and differences in their refractoriness to *Plasmodium* parasites are not clear. Genotyping TEP1 in local vector populations is, therefore, critical for monitoring changes in abundance that could explain sporozoite rates and potential malaria prevalence in varying levels of endemicities and is a potential tool for developing vector control interventions. Furthermore, information regarding the impact of vector control and environment changes on vector competence and underlying molecular mechanisms will significantly improve our understanding of malaria transmission dynamics. This study was designed to determine the distribution of TEP1 alleles circulating in *An. gambiae *sensu lato (*s.l*.) vectors in malaria-endemic regions in western Kenya.

## Methods

### Study sites and design

**Fig. 1:**
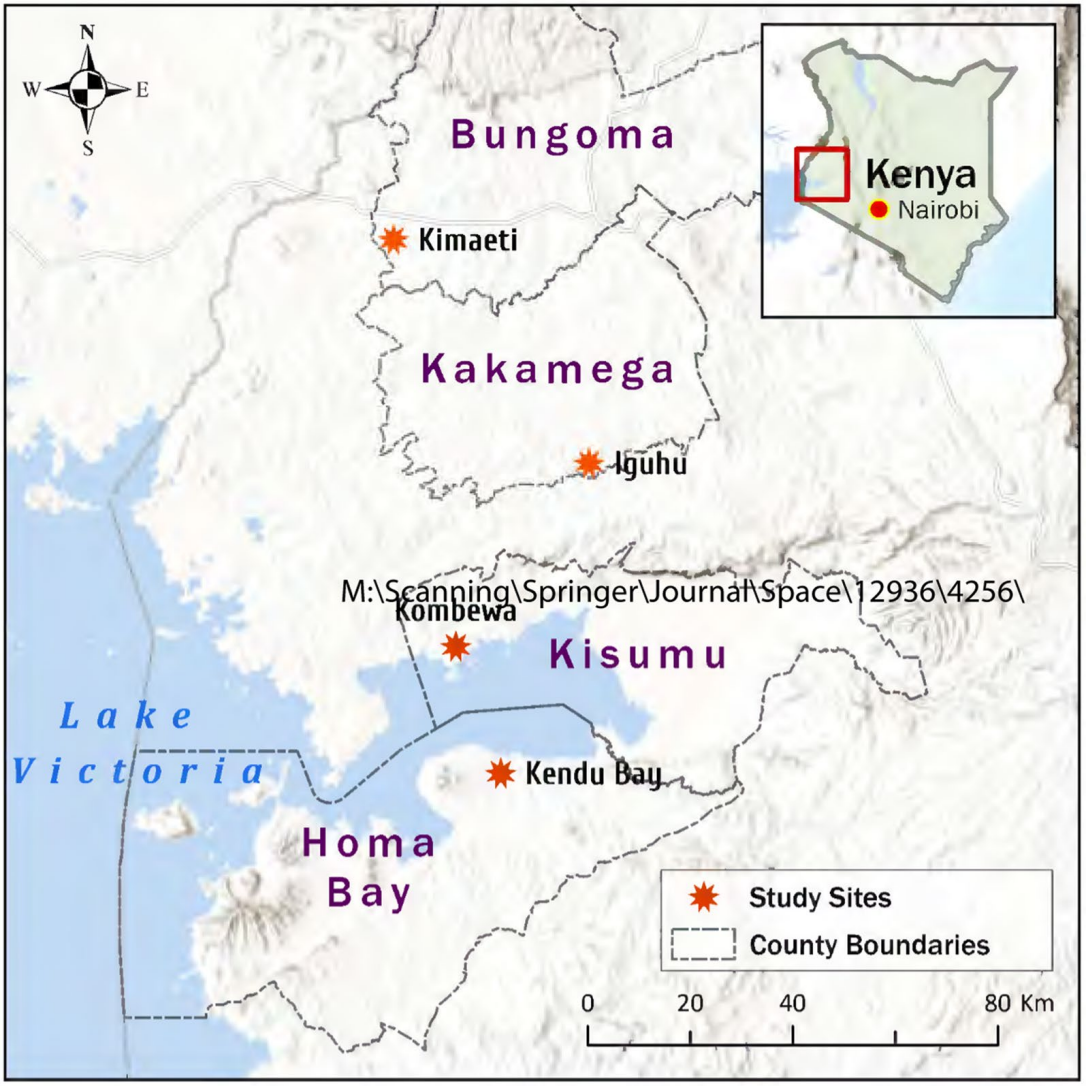
Geographical location of the mosquito collection sites in Western Kenya

This study was conducted in four counties in western Kenya namely, Bungoma, Kakamega, Kisumu, and Homa Bay (**Fig. ****1**). Two malaria epidemic-prone highland sites including Kimaeti (00.6029° N, 034.4073° E; altitude 1430–1545 m above sea level) in Bungoma, and Iguhu (34°45′E, 0°10′N; 1430–1580 m above sea level) in Kakamega, and two malaria-endemic lowland sites located around Lake Victoria; Kombewa (34°30′E, 0°07′N; 1150–1300 m above sea level) in Kisumu and Kendu Bay (34.64190°E-0.38000°S; 1134–1330 m above sea level) in Homa Bay. The climate in western Kenya consists of long and short rainy seasons that malaria transmission peaks between March to May and October to November respectively. Temperature ranges from a minimum of 14-18 ℃ to a maximum of 30-36 ℃ and average rainfall ranges between 1740 and 1940 mm annually. *Plasmodium falciparum* is the most common cause of malaria and is primarily transmitted by *An. gambiae *sensu stricto (*s.s.*)*, Anopheles funestus* and *Anopheles arabiensis* [27, 28]. The key vector control interventions are long-lasting insecticidal nets (LLINs) and indoor residual spraying (IRS) [29]. Indoor residual spray was conducted in Homa Bay County once a year in 2017 and 2018, unlike the other sampling sites.

### Adult sampling

*Anopheles* mosquitoes were collected in a cross-sectional study design using pyrethrum spray catch (PSC) from 30 randomly selected houses per site. Mosquito sampling was done during the middle of the dry season in February-March and 4 weeks after the start of the long rainy season in May–July between 2017 and 2020. Collections were conducted between 0630 and 1000 h in the morning and transported to the Sub-Saharan Africa International Center of Excellence for Malaria Research (ICEMR), Homa Bay, Kenya. Samples were stored at –20 °C in 1.5 ml Eppendorf tubes containing silica gel and assigned a unique code for further molecular processing.

### Larval sampling

Larval sampling was conducted using 350 ml standard dippers and hand pipettes [30]. A total number of dips taken from each habitat was between 5 and 20 and the presence or absence of larvae was recorded. To avoid collecting siblings from the same pool, larvae were randomly sampled from different breeding habitats. Collected larvae were labeled by habitat type and identified morphologically using the referenced keys [31]. Only *Anopheles* larvae were sorted and transported to the ICEMR insectary. The larvae were reared to adults using standardized rearing methods [32]. Emerged adults were anesthetized using chloroform and identified using the morphological key in the laboratory as described by Gillies and Coetzee to species [33, 34].

### Molecular identification of mosquito species

Genomic DNA was extracted from randomly selected single *An. gambiae* female adult using the Chelex resin (chelex^®^ -100) method following a protocol by Musapa et al. [35]. Briefly, deionized water was added into single mosquito sample tubes and ground into a uniform suspension. Phosphate buffer saline 1X and 10% saponin was then added to sample homogenates, mixed gently, and incubated at room temperature for 20 min. The suspension was then centrifuged and the supernatant discarded. The pellets were then resuspended in PBS 1X and centrifuged, supernatant discarded, and gently vortexed. The pellets were then suspended in sterile deionized water and 20% Chelex-resin suspension in deionized water. The samples were incubated at 85 °C for 10 min, centrifuged at 20,000 × g for a minute, and DNA transferred into prelabelled storage vials. *Anopheles gambiae* was identified to sibling species using polymerase chain reaction (PCR) as described by Scott et al. [36].

### Genotyping and DNA sequencing of TEP1 alleles in *Anopheles gambiae* mosquitoes

Genotyping of TEP1 alleles was performed using polymerase chain reaction-restriction fragment length polymorphism (PCR-RFLP) method as described by Gildenhard et al. [24]. Briefly, the initial PCR was conducted using Nest 1 primers - VB3 5′ GATGTGGTGAGCAGAATATGG 3′ and VB4- 5′ ACATCAATTTGCTCCGAGTT 3′ targeting 892 base pairs, followed by a second PCR performed on 5 μl of the resulting product from Nest 1 with Nest 2 primers VB1 5′ ATCTAATCGACAAAGCTACGAATTT 3′ and VB2 5′ CTTCAGTTGAACGGTGTAGTCGTT 3′ producing a final fragment length of 758 base pairs. Both PCR reaction conditions were set as denaturation at 95 °C for 3 min, 35 cycles of 94 °C for 30 s, annealing at 55 °C for 30 s, extension at 72 °C for 30 s, and a final step at 72 °C for 6 min using DreamTaq Green Master Mix (Thermo Fisher Scientific). PCR products were digested by restriction enzymes *Bam* HI, *Hind* III, or *Bse* NI (New England Biolabs Inc) (Additional file 1: Table S1) according to the manufacturer’s instructions and analyzed the result with 2.5% agarose gel electrophoresis. The TEP 1 allelic classes were then determined by fragment size of restriction enzyme digestion (Additional file 1: Table S1). A subset of samples with identified TEP1 alleles were further used for confirmatory purposes by sequencing 9 respective Nested II amplicons. Sequencing was done using 3700/3730 BigDye^®^ Terminator v3.1 Sequencing Standard kit (ABI PRISM^®^ 3700 DNA Analyzer).

### Statistical analysis

Descriptive statistical analyses were performed using GraphPad Prism v.8.0.1 Software and SPSS version 25 for Windows. Statistical significance was set at P ≤ 0.05. TEP1 allele frequencies observed heterozygosity (*Ho*), and expected heterozygosity (*He*), the inbreeding coefficients (F_IS_), departure from Hardy-Weinberg expectations were analyzed using GenEAlex version 6.053 software [37]. DNA sequences of TEP1 haplotypes were compared with published sequences. Basic Local Alignment Search Tool (BLASTN) was used to retrieve sequences from the National Center for Biotechnology Information (NCBI) database with a high similarity index to each of the haplotype sequences. The retrieved sequences with accession numbers AF291654.1, FN431783.1, FN431782.1, FN431785.1, FN431784.1, and MF098591.1 together with the identified haplotype sequences in this study were aligned. MView web-based tools [38] were used to conduct the alignment of the sequences and to calculate pairwise sequence identity and similarity. Phylogenetic analysis of the representative sequenced and GenBank retrieved TEP1 sequences was performed using MEGA 7.0 software [39]. AMOVA was used to determine the level of genetic allele differentiation among populations and within individuals. The *F*_ST_ values 0 ≤ 0.05 were interpreted as low differentiation, 0.05 ≥ 0.15 moderate differentiation and 0.15 ≥ 0.25 high levels [40].

## Results

### Species composition of *An. gambiae s.l.* across study sites

A total of 627 *An. gambiae s.l.* adults were collected and molecularly identified to sibling species based on species-specific PCR. Overall, the species identified were *An. gambiae s.s.* and *An. arabiensis* constituting 49.28% (309/627) and 50.72% (318/627) of the total samples genotyped respectively (Table 1). There was a significant difference in species abundance (*An. gambiae s.s.* versus *An. arabiensis*) in the total analy*z*ed samples (P < 0.0001) however a significant difference in species catches was only observed in Kisumu and Homa bay (P < 0.0001) (Table 1).

**Table 1:** Molecular determined species composition in western Kenya

Sampling sites	*An. arabiensis,* n (%)	*An. gambiae,* n (%)
Bungoma	39 (37.5)	65 (62.5)
Kakamega	29 (34.1)	56 (65.9)
Homa Bay	186 (82.7)	39 (17.3)
Kisumu	55 (25.8)	158 (74.2)
Grand Total	309 (49.3)	318 (50.7)

### TEP1 allele distribution in the study sites

**Fig. 2:**
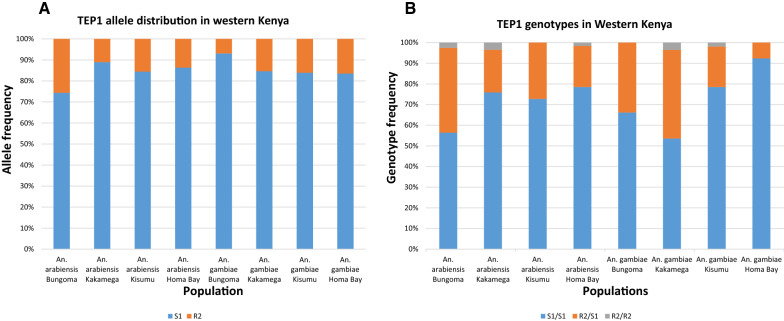
Distribution of TEP1 genotypes and alleles circulating in *An. gambiae* and *An. arabiensis* in Bungoma, Kakamega, Homa Bay, and Kisumu Counties in western Kenya

Overall, two TEP1 alleles (TEP1*S1, and TEP1*R2) were identified with average frequencies of 84.9% and 15.1%, respectively. *Anopheles arabiensis* populations from Homa Bay had the highest TEP1*S1 allele frequency (89%, 95% CI 85.8% −92.2%) which significantly differed from observed proportions in Kisumu (86.4%, 95% CI 79.8%–92.9%), Kakamega (84.5%, 95% CI 74.9%–94.1%) and Bungoma (74.4%, 95% CI 64.5%–84.3%) (Two-tailed p < 0.0001). Among *An. gambiae s.s*. populations from Bungoma displayed the highest TEP1*S1 allele frequency (93.1%, 95% CI 88.7%–97.5%) followed by Homa Bay (84.6%, 95% CI 76.4%–92.8%), Kakamega (83.9%, 95% CI 77%—90.8%), and Kisumu (83.5%, 95% CI 79.4%%–87.7%) respectively (Fig. 2A). The observed TEP1*S1 allele frequency in Bungoma significantly differed from Kakamega (two-tailed p = 0.0466) and Kisumu (two-tailed p < 0.0001). The highest TEP1*R2 allele frequency among *An. arabiensis* was observed in vector populations from Bungoma (26%, 95% CI 15.7%–35.5%) followed by Kakamega (15.5%, 95% CI 5.91%–25.1%), Kisumu (13.6%, 95% CI 7.12%–20.2%), and Homa Bay (11%, 95% CI 8.06%–14.5%). In *An. gambiae* the TEP1*R2 allele frequency was highest in populations from Kisumu and Kakamega displaying allele frequencies of 16.5%, 95% CI 12.3%–20.5% and 16.1%, 95% CI 9.16%–23%, respectively, followed by Homa Bay (15.4%, 95% CI 7.20%–23.6%) and Bungoma (7%, 95% CI 7.20%–23.6%), respectively. No significant differences in allele frequency were observed between species (P = 0.799) and between site variation (P > 0.05).

### TEP1 genotype distribution in the study sites

A total of 3 genotypes were identified in populations of *An. gambiae s.l.* in western Kenya. Out of the 3 genotypes, 2 were homozygous (TEP1*S1/S1 and TEP1*R2/R2) and 1 heterozygous (TEP1*R2/S1). Homozygote TEP1*S1/S1 and heterozygote TEP1*R2/S1 genotypes had distinct frequencies (Fig. 2B). TEP1*S1/S1 and TEP1*R2/S1 genotypes were commonly present among species in all sites at an average frequency of 71.75% and 26.61%, respectively. TEP1*R2/R2 although rare, was only present in *An. arabiensis* from Bungoma (2.6%), Kakamega (3.4%) and Homa Bay (1.6%) and *An. gambiae s.s.* from Kakamega (3.6%) and Kisumu (1.9%), but in the lowest average frequency of 1.64% (Fig. 2B). The TEP1*S1/S1 genotype was predominant followed by TEP1*R2/S1 but in low varied frequencies among species across all sampling sites. The TEP1*S1/S1 genotype frequency was highest in *An. gambiae* as compared to *An. arabiensis* from all sites except Kakamega populations that displayed higher TEP1*S1/S1 frequencies in *An. arabiensis* (75.9%) than in *An. gambiae* (53.6%) (Fig. 2B). On the contrary, the distribution of TEP1*R2/S1 genotypes was highest in *An. arabiensis* than *An. gambiae* in all sites except populations from Kakamega where higher genotype frequencies (42.9%) were observed in *An. gambiae s.s.* than in *An. arabiensis* (20.7%). The observed RFLP results for each TEP1 allele were confirmed by respective sequences upon alignment with reference sequences from the NCBI database. The TEP1*S1 and TEP1*R2 sequences had 100% identity matrix to AF291654.1 and FN431784.1 respectively. A significant difference in genotype frequency was observed among sites in *An. gambiae* populations (Fisher's exact test two-sided p-value < 0.001, n = 309), whereas no significant difference was observed among sites in *An. arabiensis* population (Fisher's exact test two-sided p-value = 0.07, n = 318).

### Evolutionary relationship based on TEP1 gene

**Fig. 3:**
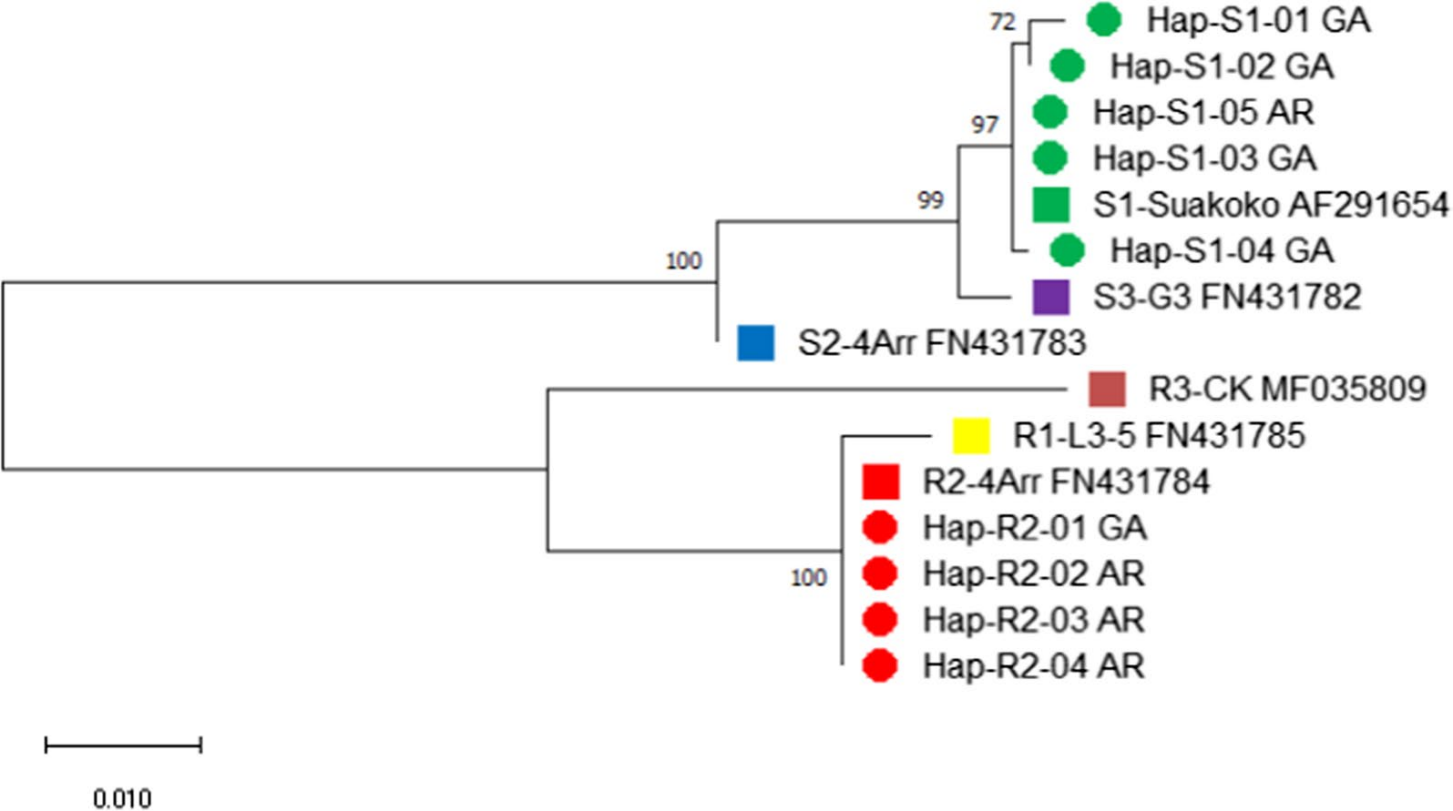
The evolutionary history was inferred by using the Maximum Likelihood method and Kimura 2-parameter model [1]. The tree with the highest log likelihood (-1872.22) is shown. The percentage of trees in which the associated taxa clustered together is shown next to the branches. Initial tree(s) for the heuristic search were obtained automatically by applying Neighbor-Join and BioNJ algorithms to a matrix of pairwise distances estimated using the Maximum Composite Likelihood (MCL) approach and then selecting the topology with a superior log-likelihood value. A discrete Gamma distribution was used to model evolutionary rate differences among sites (5 categories (+*G*, parameter = 0.7700). The rate variation model allowed for some sites to be evolutionarily invariable ([+*I*], 43.64% sites). The tree is drawn to scale, with branch lengths measured in the number of substitutions per site. This analysis involved 15 nucleotide sequences. There were a total of 873 positions in the final dataset. Evolutionary analyses were conducted in MEGA X [2]. Red and green dots indicate haplotypes identified in this study; squares with different colors represent reference haplotypes extracted from GenBank

The phylogenetic analysis of TEP1 sequences showed that alleles were clustered into susceptible and resistant groups with high bootstrap values, ranging from 72 to 100%. Out of the sequences retrieved from the gene bank, TEP1*S1 alleles identified in the study sites have a common lineage with TEP1*S1 (AF291654) from Suakoko, Liberia. TEP1*S1 evolved as a result of a mutation on the mosquito strain G3 with TEP1*S3 (FN431782) which had a close ancestral lineage with strain 4Arr that had the TEP1*S2 (FN431783) allele. TEP1*R2 from the study sites and TEP1*R1 independently evolved from TEP1*R3 (MF035809) which shared common ancestral lineage with the Susceptible (S) alleles (Fig. 3).

### Heterozygosity and departure from Hardy Weinberg Equilibrium (HWE)

The overall mean observed heterozygosity (*H*_*o*_) and expected heterozygosity (*H*_*e*_) of TEP domain in *An. gambiae s.s.* and *An*. *arabiensis* across all sites were 0.270 ± 0.035 and 0.251 ± 0.025, respectively. There were slight variations between observed heterozygosity, *H*_*o*_ ranges 0.188–0.462 in *An. arabiensis* and 0.138–0.321 in *An. gambiae s.s*. The *An. gambiae* populations from Bungoma, Kakamega, and Homa bay, and *An. arabiensis* from Bungoma and Kisumu also showed similar trends of higher observed heterozygosity than the expected with negative *F*_*IS*_ values (Table 2). A deviation was observed among *An. gambiae* from Kisumu and *An. arabiensis* from Kakamega and Homa Bay which displayed slightly higher expected heterozygosity than observed signifying the presence of inbreeding among these populations ().

**Table 2:** Genetic diversity of *An. gambiae* (GA) and *An. arabiensis* (AR) in western Kenya

Population	N	Na	*Ho*	*He*	F
AR-Bungoma	39	2.000	0.462	0.381	–0.210
AR-Homa Bay	186	2.000	0.188	0.196	0.041
AR-Kakamega	29	2.000	0.241	0.262	0.079
AR-Kisumu	55	2.000	0.236	0.236	–0.004
GA-Bungoma	65	2.000	0.138	0.129	–0.074
GA-Homa Bay	39	2.000	0.308	0.260	–0.182
GA-Kakamega	56	2.000	0.321	0.270	–0.191
GA-Kisumu	158	2.000	0.266	0.275	0.033

N represents the total number of mosquitoes sampled per study site, Na- Number of alleles per site, Ho- Observed heterozygosity, He- Expected heterozygosity, F- Fixation index

The *F*_*IS*_ showed a negative and non-significant value in *An. arabiensis* population from Bungoma (−0.210) and Kisumu (−0.004) and *An. gambiae* from Bungoma (−0.074), Kakamega (−0.191), and Homa Bay (−0.182). These results indicate a slight departure from HWE and excess of heterozygotes in these populations. The *F*_*IS*_ results for *An. arabiensis* from Homa Bay (0.041) and Kakamega (0.079) and *An. gambiae* from Kisumu (0.033) infer possible inbreeding. None of the analysed population was at HWE as all the computed values were nonsignificant (P > 0.05). The computed HWE values for *An. arabiensis* across the four localities ranged from 0.001 to 0.307 whereas for *An. gambiae* ranged 0.174 to 2.053 which was > 1.

### Population structure

The pairwise Wright's fixation index (*F*_ST_) values revealed a low genetic differentiation among *An. arabiensis* and *An. gambiae.* Zero value represented complete Panmixis between species in the subpopulations. The *F*_ST_ values ranged from no subdivision to moderate differentiation (0.000–0.036) among *An. arabiensis* from the four study sites (Table 3). A moderate differentiation in *An. arabiensis* was observed between Bungoma and Homa bay subpopulations (*F*_ST_ = 0.036). The *F*_ST_ values ranged from 0.000 to 0.022 among the *An. gambiae* subpopulations across the four regions. No population differentiation was observed between Kakamega and Homa Bay, Kisumu and Homa Bay, and Kakamega and Kisumu subpopulations (*F*_ST_ = 0). All pairwise *F*_ST_ values for *An. gambiae* and *An. arabiensis* from all regions across western Kenya demonstrated low population differentiation (0 ≤ 0.05) except *An. arabiensis* and *An. gambiae* from Bungoma that showed moderate differentiation (0.05 ≥ 0.15). The overall low levels in population structure (*F*_ST_ = 0.019) across all sites were supported by the high level of gene flow (Nm = 12.571) and low Nei’s genetic distance values (< 0.5) among the subpopulation.

**Table 3:** Pairwise comparison of *F*_ST_ among *An.gambiae* and *An. arabiensis* populations in western Kenya

Population	AR-Bungoma	AR- Homa Bay	AR-Kakamega	AR-Kisumu	GA-Bungoma	GA- Homa Bay	GA-Kakamega	GA-Kisumu
AR-Bungoma	0							
AR-Homa Bay	0.036	0						
AR-Kakamega	0.016	0.004	0					
AR-Kisumu	0.023	0.002	0.001	0				
GA-Bungoma	0.064	0.005	0.019	0.012	0			
GA-Homa Bay	0.016	0.004	0	0.001	0.018	0		
GA-Kakamega	0.014	0.005	0	0.001	0.021	0	0	
GA-Kisumu	0.013	0.006	0	0.002	0.022	0	0	0

The AMOVA results revealed that 99 percent of the observed variations in allele frequency were within each of the mosquitoes sampled (n = 627) within respective populations, and a 1% variation was observed among the eight populations, but no variations were observed among individuals (Table 4) indicating that the level of genetic differentiation between populations was very low.

**Table 4:** Analysis of molecular variance of the TEP1 gene in An. gambiae populations circulating in western Kenya

Source	*df*	SS	MS	Est. Var	%
Among Pops	7	2.306	0.329	0.001	1%
Among Individuals	619	71.992	0.116	0.000	0%
Within Individuals	627	77.000	0.123	0.123	99%
Total	1253	151.298		0.124	100%

*df*, degrees of freedom; SS, sum of squares; MS, mean squares

## Discussion

*Plasmodium falciparum* triggers an immune response in *An. gambiae* mosquitoes [41]. Following infections with *P. falciparum* in *An. gambiae*, the midgut mounts specific and nonspecific immune responses to minimize epithelial damage [42]. Interactions between specific TEP1 and leucine-rich protein complex (LRIM1 and APL1C) are important components of the mounted immune response [18, 43]. This study identified two alleles (TEP1*R2 and, TEP1*S1) in *An. gambiae* s.l from western Kenyaregions with different malaria endemicities. A high similarity index was observed among sequenced alleles that were initially identified by RFLP-PCR and sequences retrieved from NCBI. Consistent with previous reports, TEP1*R2 and TEP1*S1 were the most common identified alleles [24, 44] circulating in western Kenya and did not display a defined distribution in sampled regions implying that they are conserved and may represent ancestral alleles maintained over generations in time. Furthermore, why these alleles have been maintained in the local populations and their roles and significance in vector competence is still not clear [13, 21, 22, 45].

Low TEP1*R allele frequencies observed in these malaria-endemic areas in our study sites may be a product of selective pressures in the TEP1 gene resulting in functional variations that select.

for susceptible mosquitoes to *Plasmodium* infection [11, 44, 46] as well as encourage evolutionary processes in the TEP1 loci [21]. Additionally, western Kenya still has relatively high malaria cases according to a recent study by Ochwedo et al. [47]. Implemented vector control interventions, such as insecticide-treated nets, indoor residual spraying, and environmental factors may determine the population structure. ITNs and IRS are two commonly used vector control interventions in Africa, and they have a direct impact on vector densities and species composition [48, 49]. For example, IRS was deployed in Homa Bay to supplement the existing malaria interventions. The pre-spray period constituted 83*% An. funestus* and 16% *An. gambiae s.l.* However, consistent with this study, there was a drift in the local species composition with 99% of vectors in the post-spray period being *An. arabiensis* in the same sites [49–51]. Indoor interventions targeting *An. gambiae* complex remain the preferred method of lowering malaria transmission risk in endemic areas. *An. gambiae s.s.* is an anthropophilic indoor malaria vector [52, 53] and is susceptible to *P. falciparum,* which may explain higher TEP1*S1/S1 frequencies unlike *An. arabiensis* that is zoophilic and an outdoor dweler [47] which haboured TEP1*R2/S1 genotypes. The susceptibility rate between the three TEP1 genotypes identified among *An. arabiensis* and *An. gambiae s.s.,* would have been confirmed by examining receptivity and sporozoite prevalence, which were, however, outside the scope of this study. Understanding the underlying molecular mechanisms that determine vector competence remains important and will thereafter contribute towards developing new vector control interventions and also complement existing control methods.

The overall *F*_ST_ values for the pairwise comparison for all populations demonstrate very minimal genetic differentiation between species and sites representing the western Kenya highlands (Bungoma and Kakamega) and lowlands (Homa Bay and Kisumu) suggesting the absence of barriers across regions. This observation does not support that ongoing intervention and ecological changes impacted on allele frequency of TEP1 in the region. The low levels of genetic differentiation correspond to an effective migration index (Nm = 12.571) indicating high levels of gene flow across the sampling sites. Expected heterozygosity values were higher than the observed heterozygosity among *An. arabiensis* from Homa Bay and Kakamega and *An. gambiae* from Kisumu implies the presence of null alleles and maybe as a result of inbreeding and non-random mating of individuals within those populations (*F*_*IS*_ 0.041, 0.079, and 0.033 respectively). The insignificant deviations from HWE imply that the TEP1 loci are under strong selection and confirm other forces such as natural mutations [54, 55] and gene flow that may directly be shaping TEP1 alleles in *An. gambiae s.l.* in western Kenya. Furthermore, ecological niches contributing towards selection forces acting on genetic variations shape the population structure of the local species populations in time and hence the adaptations of these malaria vectors to available breeding habitats [56].

## Conclusion

This study reveals minimal genetic differentiation and a low population structure of the TEP1 alleles in the highland and lowland regions of western Kenya with different malaria transmission patterns. TEP1*R2 and TEP1*S1 were the most common alleles across all regions indicating that *An. gambiae* and *An. arabiensis* may have had these specific alleles before inhabiting new ecological niches. However, further studies should be carried out to investigate the implication of the current distribution of TEP1 alleles on vector competence and sporozoite rates in mosquito populations and the importance of TEP1 surveillance for malaria control.

## Supplementary Information

**Additional file 1: Table S1.** Restriction enzyme digestion of PCR products of TEP1 gene in *An. gambiae *and* An. arabiensis* in western Kenya

## Data Availability

The data sets generated and analyzed during this study are available from the corresponding authors on reasonable requests.

## Abbreviations

RT-PCR: Real-time polymerase chain reaction
LLIN: Long-lasting insecticidal net
IRS: Indoor residual spraying
DBS: Dried blood spots
DNA: Deoxyribonucleic acid
PCR: Polymerase chain reaction
