# Supplementary material for: Molecular characterization and genotype distribution of thioester-containing protein 1 gene in Anopheles gambiae mosquitoes in western Kenya
Source: Malar J. 2022 Aug 10;21:235. doi: 10.1186/s12936-022-04256-w (PMC9364548; doi:10.1186/s12936-022-04256-w)
Supplement: Supplementary file 1 — Additional file 1: Table S1. Restriction enzyme digestion of PCR products of TEP1 gene in An. gambiae and An. arabiensis in western Kenya [file 12936_2022_4256_MOESM1_ESM.docx]

**S1 Table.** Restriction enzyme digestion of PCR products of TEP1 gene in An. gambiae and An. arabiensis in western Kenya

| **TEP1 genotype** | **Fragment sizes (bp)** | | |
| --- | --- | --- | --- |
|  | ***Bam*HI** | ***Hind*III** | ***Bse*NI** |
| **S1/S1** | 758 | 656 | 537 |
|  |  | 102 | 221 |
| **S1/S2** | 758 | 656 | 758 |
|  |  | 102 | 537 |
|  |  |  | 221 |
| **R2/S1** | 758 | 758 | 657 |
|  | 399 | 656 | 221 |
|  | 359 | 102 | 537 |
|  |  |  | 101 |
| **R2/R2** | 399 | 758 | 657 |
|  | 359 |  | 101 |
